# Delay to surgery in acute perforated and ischaemic gastrointestinal pathology: a systematic review

**DOI:** 10.1093/bjsopen/zrab072

**Published:** 2021-09-03

**Authors:** V Murray, J R Burke, M Hughes, C Schofield, A Young

**Affiliations:** 1 The University of Leeds Medical School, Leeds, UK; 2 The John Golligher Colorectal Surgery Unit, St. James’s University Hospital, Leeds, UK; 3 Leeds Institute of Biomedical & Clinical Sciences, Clinical Sciences Building, St James’s University Hospital, Leeds, UK; 4 Department of Anaesthetics, St James’s University Hospital, Leeds, UK; 5 Department of Pancreatic Surgery, St James’s University Hospital, Leeds, UK

## Abstract

**Background:**

Patients with acute abdominal pathology requiring emergency laparotomy who experience a delay to theatre have an increased risk of morbidity, mortality and complications. The timeline between symptom onset and operation is ill defined with international variance in assessment and management. This systematic review aims to define where delays to surgery occur and assess the evidence for interventions trialled across Europe.

**Methods:**

A systematic review was performed searching MEDLINE and EMBASE databases (1 January 2005 to 6 May 2020). All studies assessing the impact of time to theatre in patients with acute abdominal pathology requiring emergency laparotomy were considered.

**Results:**

Sixteen papers, involving 50 653 patients, were included in the analysis. Fifteen unique timepoints were identified in the patient pathway between symptom onset and operation which are classified into four distinct phases. Time from admission to theatre (1–72 hours) and mortality rate (10.6–74.5 per cent) varied greatly between studies. Mean time to surgery was significantly higher in deceased patients compared with that in survivors. Delays were related to imaging, diagnosis, decision making, theatre availability and staffing. Four of five interventional studies showed a reduced mortality rate following introduction of an acute laparotomy pathway.

**Conclusion:**

Given the heterogeneous nature of the patient population and pathologies, an assessment and management framework from onset of symptoms to operation is proposed. This could be incorporated into mortality prediction and audit tools and assist in the assessment of interventions.

## Teaser

Whilst delay to theatre in patients with acute abdominal pathology requiring emergency laparotomy is associated with an increased risk of mortality, there is wide variation in the definition and measurement of time delays prior to emergency surgery with few studies exploring interventions to reduce delays. An assessment and management framework from onset of symptoms to operation is proposed.

## Introduction

Emergency laparotomies for acute abdominal pathology carry a high mortality rate which remained static at 9.6 per cent between 2016 and 2018 in the UK^1^, with studies in Europe and the US suggesting a 30-day mortality of 9.6–18.5 per cent^1–3^. These figures are far higher than the 1–2 per cent risk seen in elective laparotomies across all specialties^4^, due to the severity of acute illness associated with patients requiring emergency laparotomies. In the UK, the National Confidential Enquiry into Patient Outcome and Death (NCEPOD) urgency classification is used as a framework for timeliness of arrival in theatre. Of the few hospitals in the UK that have implemented ‘acute abdomen’ pathways, only 36.3 per cent include guidance on timing to surgery^5^.

Emergency surgical patients are classified as requiring immediate (under 2 hours) or urgent 2A (2–6 hours) surgical intervention (as opposed to urgent 2B: within 6–18 hours)^6^. Whilst the target in the UK is for 85 per cent of patients to arrive in theatre within the appropriate timeframe as a minimum standard, this has not been achieved in the latest reported years (82.4 per cent in 2018, which is unchanged from 2016). Over a quarter of the patients requiring immediate surgery (within 2 hours) do not arrive in theatre within the recommended time^1^. In the UK, around 25 000–35 000 patients in this cohort each year have intra-abdominal sepsis, a leading cause of deterioration and death. Of those that require surgical source control, patients who receive intervention beyond the NCEPOD timeframe have an almost three percentage points higher mortality risk than those operated on within the recommended time (15.9 per cent compared with 13 per cent)^1^. The Surviving Sepsis Guideline recommends that rapid intervention for source control should occur within 12 hours^7^ and the Royal College of Surgeons recommends that source control should not be delayed over 6 hours for either observation or resuscitation^8^.

Delays to theatre inevitably increase the risk of sepsis, deterioration, failure to rescue and death^9^. Despite this, the timeline between symptom onset and emergency laparotomy is ill defined with variance in the assessment and management. There appears to be no universally adopted standardized pathway for this patient cohort. The aim of this systematic review is to define a pathway to identify where patients with acute abdominal pathology requiring emergency laparotomy experience delay to theatre, and to evaluate the evidence for previously reported interventions trialled across Europe to reduce delays.

## Methods

The protocol for this review was undertaken in accordance with the preferred reporting items for systematic reviews and meta-analyses guidelines for protocols (PRISMA-P)^10^. The review was registered with PROSPERO on 4 June 2020 (registration number: CRD42020185070).

### Eligibility criteria

All interventional, cohort, case-control and cross-sectional studies that investigated the impact of delay to theatre or interventional studies across Europe to reduce delay were considered. The population of included studies were adults (age 18 years and older) with emergency laparotomy required under NCEPOD urgency classifications immediate or urgent. The following life-threatening pathologies requiring emergency laparotomy were included: perforated gastrointestinal viscus including perforated bowel, perforated peptic ulcer, diverticular perforation, perforated closed loop obstruction and colonic perforation; ischaemic gastrointestinal viscus including ischaemic bowel, ischaemic closed loop obstruction and acute mesenteric ischaemia. Included articles were published between January 2005 (introduction of NCEPOD classification) and October 2020^11^. The outcome measure was reported mortality rate.

Studies including endovascular or re-laparotomy procedures were excluded. Appendectomy, cholecystectomy and simple hernia repairs were excluded as they are associated with less severe illness and mortality rate. Subjects admitted initially for conservative therapy and those with renal, vascular, gynaecological or obstetric, trauma and iatrogenic pathology were also excluded. Conference abstracts and studies not in English language and not in humans were excluded.

### Study selection

The following electronic databases published between 1 January 2005 and 6 May 2020 were searched: MEDLINE and EMBASE. Citations and reference lists of selected studies were reviewed to identify missed articles. The search was undertaken on 7 May 2020 and updated on 1 October 2020. Dates were restricted to between 2005 and 1 October 2020 to align with the introduction of the NCEPOD classification system introduced in December 2004^11^. Conference abstracts were excluded due to high risk of incomplete data. Supplementary material online details the full search strategy.

Studies were selected via a staged review of titles and abstracts, followed by full-text review. Abstracts were retrieved by one investigator (V.M.) and cross-checked by another investigator (M.H.). The search-identified abstracts and those from citations and reference lists were independently screened by V.M. and M.H. to identify studies that met the inclusion criteria. Discrepancies were settled through discussion with J.B.

### Data-collection process

Data were extracted into a spreadsheet (Microsoft Excel; Microsoft, Redmond, Washington, USA). The following baseline data were extracted from each study: study title, authors and date; patient characteristics (acute pathology/pathologies), study design, sample size, study setting (country), definition of surgical delay, time points from symptom onset to surgery, time point intervals, mortality rate, and causes for delay.

### Outcomes

The primary outcome was mortality rate, defined as mortality within 30, 60 and 90 days of surgery. Time points from symptom onset to surgery and the effectiveness of any intervention, trialled to improve delay from symptom onset to emergency operation, on reducing delays to theatre and reducing mortality were secondary outcomes.

### Risk of bias in individual studies

The methodological quality of the studies and risk of bias were assessed by two independent reviewers (V.M. and J.B.) using the 16-item Quality Assessment Tool for Studies with Diverse Designs (QATSDD), chosen for its reliability in assessing a diverse range of study designs. This was completed after study selection. QATSDD includes a 16-item assessment tool which generates a methodology quality score as a percentage for each study^12^.

### Data synthesis

Quantitative data synthesis was not performed given the marked heterogeneity of study designs included in the review. A narrative synthesis approach was therefore chosen to summarize the diverse range of selected studies, following the European Social Research Council Guidance on the Conduct of Narrative Synthesis in Systematic Reviews^13^. The studies were then grouped into interventional studies and observational studies. The results within these defined groups were tabulated to highlight designs and findings. The evidence was then synthesized into data on the structure and coordination of pathways and then a timeline of prehospital, preimaging, decision and preoperative phases between symptom onset and knife-to-skin, to provide a framework on which to identify where delay occurs.

## Results

**Fig. 1:**
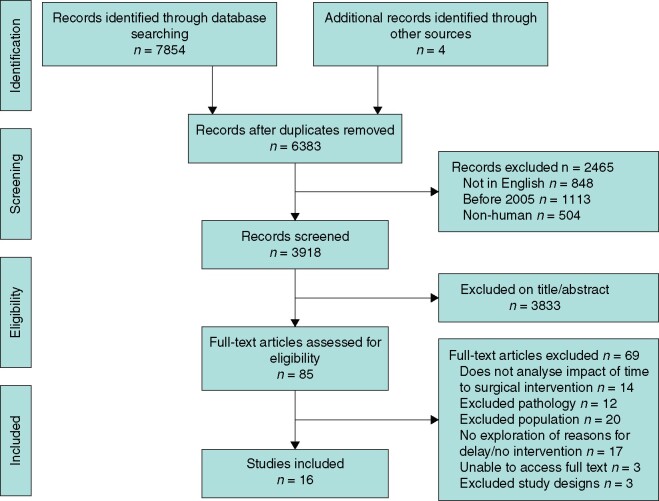
PRISMA flow diagram

The initial search generated 7854 papers. After screening, 84 were assessed on full text for eligibility (*Fig. 1*). Of these, 16 publications were included in the final analysis (*Tables 1* and *2*). The results show papers relating to whole pathway analysis and are then broken down into four chronological time periods along the patients’ journey from symptom onset to knife-to-skin.

**Table 1:** Interventional studies

No.	Title	Authors, country, year	Study design/sample size	Study question/aim	Intervention/control (no. of components)	Pathology	Main findings
**1**	Multicentre trial of a perioperative protocol to reduce mortality in patients with peptic ulcer perforation	Møller *et al*.^14^ Denmark 2011	Externally controlled multicentre trial (PULP trial) *n* = 2619	To evaluate a multimodal and multidisciplinary perioperative care protocol on mortality in patients with PPU	Intervention protocol (11): 1. evaluation and risk stratification; 2. minimization of surgical delay – surgery within 6 h of admission; 3. early antibiotics; 4. blood tests and ECG; 5. respiratory and circulatory stabilization in an HDU; 6. antisecretory therapy; 7. nutrition and fluids after surgery; 8. analgesia; 9. early mobilization; 10. prevention of atelectasis and other complications; 11. monitoring	PPU	Mortality was reduced from 27 to 17% applying a multimodal care protocol
**2**	Use of a pathway quality improvement care bundle to reduce mortality after emergency laparotomy	Huddart *et al*.^15^ UK 2014	Multicentre Emergency Laparotomy Pathway–QI Care (ELPQuIC) bundle *n* = 726	To compare 30-day mortality after emergency laparotomy before and after the ELPQuiC bundle	Intervention protocol (5): EWS; 2. early antibiotics; 3. interval between decision and operation less than 6 h; 4. goal-directed fluid therapy; 5. postoperative intensive care Control: Each hospital submitted ELPQuiC baseline data before implementation on consecutive patients for a minimum of 3 months before the start of the project	Mixed GI pathology	Increased lives saved per 100 patients treated, from 6⋅47 in the baseline interval to 12⋅44 after ELPQuiC (*P* < 0⋅001). The overall case mix-adjusted risk of death decreased from 15.6 to 9.6%
**3**	Multidisciplinary perioperative protocol in patients undergoing acute high-risk abdominal surgery	Tengberg *et al*.^16^ Denmark 2017	Prospective single-centre controlled trial *n* = 1200	To evaluate a standardized multidisciplinary perioperative protocol in AHA surgery	Intervention protocol (9): continuous staff education; 2. consultant-led care; 3. early resuscitation and high-dose antibiotics; 4. surgery within 6 h of indication to operate; 5. perioperative stroke volume-guided haemodynamic optimization; 6. intermediate level of care for the first 24 h after surgery; 7. standardized analgesia; 8. early postoperative ambulation; 9. early enteral nutrition Control: historical cohort	Perforated viscus; intestinal obstruction; bowel ischaemia; peritonitis	The multidisciplinary perioperative protocol was associated with a significant reduction in postoperative mortality in patients undergoing AHA surgery
**4**	EPOCH trial: Effectiveness of a national QI programme to improve survival after emergency abdominal surgery	Peden *et al*.^17^ UK 2019	A stepped-wedge cluster randomized trial Single blinded *n* = 15 837	To evaluate the EPOCH care pathway to improve survival for these patients	Intervention protocol (37): 11. surgery within 6 h of decision to operate	Peritonitis; perforation; intestinal obstruction; haemorrhage; ischaemia; abdominal infection; other	There was no survival or LOS in hospital benefit from a QI programme to implement a care pathway for patients undergoing emergency abdominal surgery
**5**	Evaluation of the collaborative use of an evidence-based care bundle in emergency laparotomy	Aggarwal *et al*.^18^ UK 2019	Multicentre QI trial of the ELC bundle *n* = 14 809	To assess whether the ELC care bundle improved mortality, LOS, and standards of care	Intervention protocol (6): blood lactate measurement; 2. early review and treatment for sepsis; 3. transfer to the surgery within 6 h of decision to operate; 4. goal-directed fluid therapy, ICU after surgery; 5. senior MDT clinicians in the decision; 6. perioperative care. Control protocol: baseline data collected for 15 months prior to interventional period	Mixed GI pathology	Unadjusted mortality rate decreased from 9.8% at baseline to 8.3% in year 2, and P-POSSUM risk-adjusted 30-day mortality from 5.3 to 4.5% following ELC respectively

PPU, perforated peptic ulcer; ECG, electrocardiogram; HDU, high dependency unit; QI, quality improvement; EWS, early warning score; GI, gastrointestinal; AHA, acute high-risk abdominal; LOS, length of stay; ELC, Emergency Laparotomy Collaborative; ICU, intensive care unit; MDT, multidisciplinary team.

**Table 2:** Observational studies

No.	Title	Authors, country, year	Study design/sample size	Study question/aim	Pathology	Main findings
**6**	Surgical delay is a critical determinant of survival in perforated peptic ulcer	Buck *et al*.^19^ Denmark 2013	National prospective cohort study *n* = 2668	To evaluate the adjusted effect of hourly surgical delay on survival after PPU	PPU	Every hour of delay from admission to surgery was associated with an adjusted 2.4% decreased probability of survival compared with the previous hour
**7**	Quality-of-care initiative in patients treated surgically for perforated peptic ulcer	Møller *et al*.^20^ Denmark 2013	National prospective cohort *n* = 2989	To analyse the results of a nationwide QI initiative to reduce preoperative delay, and improve perioperative monitoring and care for patients with PPU	PPU	The initiative was associated with reduced preoperative delay. A non-significant improvement was seen in 30-day mortality
**8**	Time from admission to initiation of surgery for source control is a critical determinant of survival in patients with gastrointestinal perforation with associated septic shock	Azuhata *et al*.^21^ Japan 2014	Single-centre prospective cohort study *n* = 154	To demonstrate statistically the relationship between time from admission to initiation of surgery and 60-day outcome	GI perforation with septic shock	Time from admission to initiation of surgery was significantly associated with 60-day outcome. The survival rate fell as surgery initiation was delayed and was 0% for times greater than 6 h (adjusted OR 0.29 per hour delay)
**9**	Increased mortality in the elderly after emergency abdominal surgery	Svenningsen *et al*.^22^ Denmark 2014	Single-centre retrospective cohort study *n* = 131	To evaluate the relation between preoperative delay and mortality in surgical patients undergoing primary emergency laparotomy	Intestinal obstruction; perforated viscus; emergency laparotomy or laparoscopy within 24 h	No association between time to operation exceeding 6 h and postoperative mortality was found. The only variable found to be significantly associated with higher mortality was age >75 years
**10**	The impact of early surgical intervention in free intestinal perforation: a time-to-intervention pilot study	Hecker *et al*.^23^ Germany 2015	Single-centre retrospective cohort pilot study *n* = 76	Time-to-intervention pilot study to investigate if surgical source control in the very early phase of early goal-directed sepsis therapy is of benefit for surgical intensive care patients	Intestinal perforation	The overall survival was 80% for study group I (intervention within 3 h) and decreased to 75% for group II (intervention within 3–9 h) and 73% in group III (intervention >9 h but the majority within 12 h). Early surgical intervention tends to result in lower rates of peritonitis (group I 88% *versus* group II 92% *versus* group III 100%)
**11**	Association between surgical delay and survival in high-risk emergency abdominal surgery. A population-based Danish cohort study	Vester-Andersen *et al*.^24^ Denmark 2016	Multi-centre prospective cohort study *n* = 2803	To evaluate the association between surgical delay by hour and mortality in high-risk patients undergoing emergency abdominal surgery	Mixed GI pathologies	Each hour of surgical delay beyond hospital admission was associated with a median decrease in 90-day survival of 2.2% but no statistically significant association between surgical delay by hour and 90-day mortality was shown
**12**	Factors associated with in-hospital death in patients with acute mesenteric artery ischemia	Élthes *et al*.^25^ Romania 2018	Single-centre retrospective cohort study *n* = 50	To assess the factors associated with mortality in patients with AMI, emphasizing the importance of an early diagnosis and a prompt surgical intervention to avoid lesion progression	AMI: arterial, venous, non-occlusive, mechanical	Increased mortality rates with longer periods of stay in the ED for diagnostic procedures until surgical intervention. Total elapsed time from ED presentation to the start of surgery (mean hours): 9.10 h in deceased group *versus* 5.57 h in survivors
**13**	Choice of first emergency room affects the fate of patients with acute mesenteric ischaemia: the importance of referral patterns and triage	Lemma *et al*.^26^ Finland 2019	Single-centre retrospective cohort study *n* = 81	To analyse the factors affecting delay in patients with AMI, with special focus on the pathways to treatment	AMI	In a non-surgical ED, the time to surgical operation was around 15 h and mortality 75%, compared with 10 h and 50% mortality if the first ED was surgical. The first specialty that the patient encounters seems to be crucial for both delayed management and early survival of AMI
**14**	Mortality for emergency laparotomy is not affected by the weekend effect: a multicentre study	Nageswaran *et al*.^27^ England and Wales 2019	Retrospective cohort – NELA *n* = 1717	This study examines whether a weekend effect exists for patients who undergo emergency laparotomy using NELA data	Perforation; peritonitis; small bowel obstruction; ischaemia; abdominal abscess; sepsis; haemorrhage; incarcerated hernia; colitis; other	There was a statistically significant shorter time to theatre (26.5 h *versus* 24.1 h, *P* = 0.020) for patients who underwent surgery on weekends. Mortality for weekdays and weekends was similar at 12.5 *versus* 12.8%
**15**	Delay in source control in perforated peptic ulcer leads to 6% increased risk of death per hour: a nationwide cohort study	Boyd-Carson *et al*.^28^ UK 2019	Prospective cohort study (from NELA)	To evaluate the potential relationship between hourly delay from admission to surgery and postoperative mortality in patients with PPU	PPU	90-day mortality rate increased as time to theatre rose by 3% per hour delay to theatre. In patients who were physiologically shocked (*n* = 334), there was an increase of 6% per hour
**16**	Delay in transit: a review of the quality of care provided to patients aged over 16 years with a diagnosis of acute bowel obstruction	NCEPOD^5^ UK 2020	Retrospective questionnaire review and case note review *n* = 3809	To highlight areas where care could be improved in patients who were admitted to hospital and had a diagnosis of acute bowel obstruction	Bowel obstruction	Significant delays were found in imaging, diagnosis, decision making, and availability of operating theatres 6 of 31 (19.4%) patients, for whom there was a delay to surgery, died during the admission, compared with 8 of 116 (6.9%) patients, for whom there was no delay to surgery and who died

PPU, perforated peptic ulcer; QI, quality improvement; GI, gastrointestinal; OR, odds ratio; AMI, acute mesenteric ischaemia; ED, emergency department; NELA, National Emergency Laparotomy. Audit.

### Study characteristics

There were six single-centre and 10 multicentre studies involving 50 653 patients: four controlled trials, one stepped-wedge randomized trial, 10 cohort studies and one questionnaire and case notes review. They range in size from 50 to 15 837 patients, with nine located in Europe, six in the UK and one in Japan.

### Whole-pathway analysis

One study reported on predefined acute abdominal pathways. The NCEPOD transit to theatre report^5^ found that 28 of 169 (16.6 per cent) hospitals in the UK had a specific pathway for acute bowel obstruction, and 63 of 169 (37.3 per cent) had a general acute abdomen pathway. Of the 91 hospitals with predefined pathways, guidelines on time to treatment decision were present in 22 (24.2 per cent) and time to surgery in 33 hospitals (36.3 per cent)^5^. A priority grading system for emergency surgery was present in 120 of 166 hospitals (72.3 per cent) and 79 of 164 (48.2 per cent) had a coordinator to facilitate optimal utilization of this limited resource^5^. Overall, this report found 126 individual patients with acute bowel obstruction experienced delay(s) to theatre (42.8 per cent), including delay in recognizing acute bowel obstruction (44 of 283 patients), surgical assessment (33 of 277), imaging (57 of 276), diagnosis (51 of 285), decision making (42 of 281) and surgery (15 of 173). Patients on a predefined acute bowel obstruction pathway were considerably less likely to experience delays in comparison with patients not on a pathway (2.6 *versus* 12.4 per cent respectively)^5^. In the opinion of the case reviewers, the clinical outcome was affected by delay in over a quarter of the patients concerned (34 of 126)^5^. Overall, 6 of 31 (19 per cent) patients who experienced a delay to surgery died during their admission, compared with 8 of 116 (6.9 per cent) of those where no delay occurred^5^.

This is supported by an 11-component perioperative interventional study by Moller and colleagues (2011)^14^. Here, they achieved surgery within 6 hours of admission in 63.2 per cent of cases (74 of 117 patients). Whilst the range overall in time to intervention (TTI) was 1–72 hours, the median TTI in the interventional group was 5 hours. This corresponded to a 17.1 per cent mortality rate in the interventional group, compared with a 27 per cent mortality rate, on average, in the three control cohorts^14^. Conversely, Svenningsen and co-workers (2014)^22^ found no association between time to emergency laparotomy exceeding 6 hours and postoperative mortality in their retrospective analysis, with a median of 8.9 hours from admission to theatre and only 35.1 per cent of patients reaching theatre within 6 hours. The only variable found to be significantly associated with higher mortality was age (75 years or above). The median overall TTI in patients where a specific plan for preoperative optimization was shorter, at 7.4 hours, compared with 9.5 hours in patients without a specific plan for optimization^22^.

Several studies suggest the time from admission to theatre for source control is a critical determinant for survival in acute abdominal pathology. Élthes and colleagues (2018)^25^ compared deceased patients with survivors in their cohort study, finding a mean time from admission to theatre of 9.10 hours compared with 5.57 hours respectively. Four studies suggested a reduced survival of 2.2–3 per cent with each additional hour of delay from admission to surgery^19^^,^^21^^,^^24^^,^^28^. Boyd-Carson and colleagues (2019)^28^ found patients with perforated peptic ulcers (PPUs) presenting in physiological shock had an increase in mortality rate of 6 per cent per hour of delay, regardless of age or co-morbidities. Hecker and co-workers (2015)^23^ found a trend of reduced peritonitis and mortality when time to surgical intervention was under 3 hours, although their results were not statistically significant. Similarly, Moller and colleagues (2013)^20^ found the initiation of theatre within 6 hours was associated with a non-statistically significant reduced 30-day mortality rate.

**Fig. 2:**
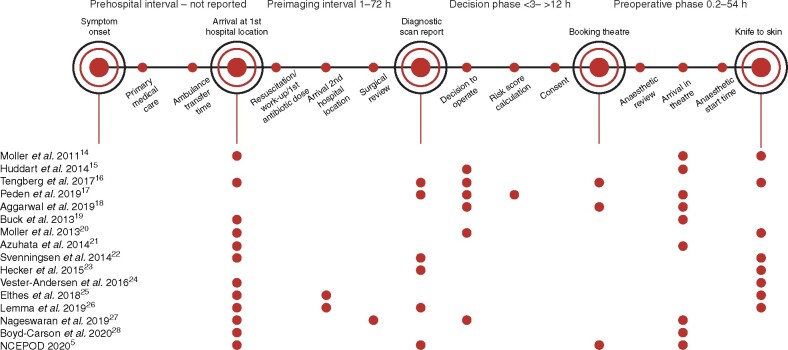
Symptoms2Surgery framework applied to included studies. Red dots indicated time points reported in each study

This systematic review identified 15 unique timepoints assessed in the emergency laparotomy pathway from the time of symptom onset to knife-to-skin (*Fig. 2*). There is currently no standardized methodological approach to study these various processes and their timings, but they could be categorized into four distinct phases: prehospital, preimaging, decision, and preoperative. This provides a framework for analysing the existing literature (*Fig. 2*) and a structure in which to assess the efficacy of interventions on certain phases of the pathway.

### Prehospital phase

No studies investigating the impact of time delay in the prehospital phase met the criteria for inclusion.

### Preimaging phase

The preimaging phase was assessed in one study by Lemma and colleagues (2019)^26^. They determined an interquartile range of 2.7–10.6 hours from emergency department (ED) presentation to computed tomography (CT) interpretation (median 5.5 hours) and 2.8–12.9 hours from ED to diagnosis (median 6.5 hours). This corresponded to an overall median interval from admission to theatre of 12.6 hours. When comparing TTI and 90-day mortality of patients presenting to an ED with surgeons on site or not, they identified the TTI was shorter (10 *versus* 15 hours) and the mortality lower (50 *versus* 75 per cent)^26^. During the investigation period, the NCEPOD report^5^ (2020) found unnecessary delays awaiting imaging in 57 of 276 patients (20.7 per cent), 35 of 57 (61.4 per cent) of whom subsequently had a delayed diagnosis. This compared with only 14 of 219 (6.1 per cent) patients having a delayed diagnosis when there was no delay in imaging^5^. Élthes and co-workers (2018)^25^ found that, regarding acute mesenteric ischaemia (AMI), the more prolonged the investigation period in the ED, the greater the lesion progression. This was directly associated with an increase in mortality rate.

### Decision phase

Of the 41 of 258 (15.9 per cent) patients that experienced delays in consultant review in the NCEPOD study^5^, this led to a delay in diagnosis in 13 of 31 (40.6 per cent) patients, compared with only 23 of 147 (15.6 per cent) patients seen by a consultant in a timely manner who subsequently had a delayed diagnosis.

The following interventional trials included an interval of 6 hours or less from decision-to-operate to surgery as one of a multi-element protocol. Huddart and colleagues (2014)^15^ introduced the Emergency Laparotomy Pathway Quality Improvement Care (ELPQuiC) bundle, a five-point interventional bundle. The risk-adjusted 30-day mortality rate dropped from 15.6 to 9.6 per cent with implementation of the bundle, although this did not reach statistical significance. This equates to 5.97 more lives saved per 100 patients treated^15^. Similarly, Aggarwal and co-workers (2019)^18^ conducted a large interventional trial across 28 hospitals, implementing a six-point care bundle. A reduction in the median P-POSSUM risk-adjusted mortality at 30-days was identified, from 5.3 per cent at baseline to 4.5 per cent with implementation of the care bundle, alongside a gradually increased rate of patients reaching surgery within 6 hours (from 77.2 per cent at baseline to 80.8 per cent at the end of the second year)^18^. Tengberg and colleagues (2017)^16^ trialled a nine-element protocol in their single-centre trial in acute high-risk abdominal surgery patients. Although a reduced 30-day and 180-day mortality rates were found in the interventional group (21.8 per cent in the control cohort compared with 15.5 per cent in the interventional cohort, and 29.5 per cent compared with 22.2 per cent respectively), no substantial change achieving surgery within the 6-hour target was met (29.1 per cent in the control cohort *versus* 26.5 per cent in the interventional cohort). The EPOCH trial, a stepped-wedge cluster randomized trial, involved a 37-element protocol. Although an improvement in the median time to theatre from 5 (i.q.r. 2.1–16.8) to 4.3 hours (i.q.r. 2.0–15.3) was found, the 90-day mortality was identical at 16 per cent in both cohorts^17^.

Hecker and colleagues (2015)^23^ conducted a retrospective TTI pilot study to investigate if surgical source control in the very early phase of early goal-directed sepsis therapy improves outcomes in patients with intestinal perforation. They demonstrated an 80 per cent survival rate of patients in the early intervention group (surgery within 3 hours) *versus* 73 per cent in the late intervention group (surgery beyond 3 hours, but predominantly within 12 hours). They found early surgical intervention results in lower rates of peritonitis (group I 88 per cent *versus* group II 92 per cent *versus* group III 100 per cent), which aligns with the differences in mortality seen^23^.

### Preoperative phase

Following diagnosis in the NCEPOD study^5^, 72 of 368 (19.6 per cent) patients experienced a delay in access to surgery. Of these 72 patients, 38 (52.8 per cent) had delays due to non-availability of theatre and 34 (47.2 per cent) had delays due to non-availability of an anaesthetist. Although 80 per cent of hospitals had a dedicated emergency theatre, this is typically a single emergency theatre (107 of 127 hospitals) which is shared with other specialties^5^. Élthes and colleagues (2018)^25^ found a mean time of 1.77 *versus* 1.42 hours in deceased *versus* survivors from arrival in surgery to knife-to-skin.

## Discussion

Delays occur throughout the pathway from symptom onset to knife-to-skin in patients with perforated or ischaemic abdominal pathology. Thirteen of the 16 studies reviewed demonstrate that delay to surgery increases the mortality rate. This is the first review to define systematically a pathway of time points where delays occur. *Figure 2* illustrates the significant proportion of time intervals that have not been analysed in the literature thus far. The NCEPOD recommend that to minimize delays to diagnosis and treatment in acute bowel obstruction, time points that should be audited include: from arrival to CT scan, from arrival to diagnosis, and from decision to operate to the start of anaesthesia^5^. This review also shows that surgery within 6 hours of admission (1 study) or decision to operate (3 studies) increases the survival of patients with life-threatening abdominal pathology, evidenced by four of five interventional studies reviewed.

In particular, the prehospital phase in emergency general surgery is ill defined, as such there were no studies identified which reported on delays within this phase. This may be due to these data not being collected routinely on hospital notes. However, this phase is arguably an aspect of the pathway that must be addressed in future studies to acknowledge the widely variable presentation of this patient cohort and to improve engagement with primary and prehospital care. Access to social networks and healthcare provision probably has an impact on the timeliness of presentation initially as well as on discharge. Although this has not been explored in this systematic review, it is an important area to consider in future research.

Although the interventional studies were homogeneous in terms of implementing a 6-hour time frame to theatre as part of their protocol, this was always part of a multiple-element protocol, making it hard to isolate one particular intervention’s effect on the outcome. Furthermore, implementing change takes time. Aggarwal and co-workers (2019)^18^ observed a gradual reduction in the mortality rate, concurrent with a gradual increase in patients reaching surgery within 6 hours. However, this improvement in TTI was only achieved in the last year of their 2-year project, suggesting that these changes are most effective once they become established in practice. The results from the EPOCH trial^29^, the largest interventional trial, demonstrating no improvement in survival with the implementation of the care pathway, are surprising as most of the papers have shown that reducing delays increases survival. However, the study group report that despite good engagement, staff had limited time and resources which led to only modest implementation, adaptation and variation of the protocol between hospitals. The trial group concluded that implementing such an extensive care pathway was more complex than expected which reiterates that such big structural changes may take longer and be more challenging than expected^17^.

Standardized protocol-based pathways for trauma have been extensively studied and implemented worldwide alongside training courses, such as the advanced trauma life support, due to the recognition that outcomes of critically unwell patients can be dependent on time-critical interventions. Quality standards are therefore in place, such as trauma patients receiving a CT within 1 hour of arrival to the ED^30^. With an acute abdominal pathology mortality rate of up to 74 per cent^25^^,^^26^, there is a convincing argument that fast-track pathways should be implemented similarly for emergency abdominal surgery.

These recommendations are strongly in line with the Enhanced Recovery After Surgery (ERAS) Society’s recently published guideline on perioperative care for emergency laparotomy^31^. It recommends source control with surgery or interventional radiology should be carried out for all patients with septic shock within 3 hours, and within 6 hours for all patients with sepsis without septic shock. The guideline states that a CT scan should be performed promptly if indicated but acquiring it should not delay surgery if the surgery is very urgent. It further points out that resuscitation must go hand in hand with diagnostic interventions and preparations for surgery, including ensuring the availability of a theatre. The NCEPOD guidance on time targets to theatre, although sensible and pragmatic, appears to be based on minimal evidence. If a framework from onset of symptoms to surgery were adopted as part of a national audit, timing could be monitored with more accurate recommendations made to help achieve the recommendations set out in the ERAS guideline. This could lead to a fast-track pathway for patients with life-threatening abdominal pathology^30^.

Limitations of this review include significant heterogeneity between the studies in design, scope and how study parameters were defined. There is also a variety of pathologies included. While the breadth of pathologies was chosen to ensure a comprehensive picture of the problematic intervals within the pathway to theatre for patients, some abdominal pathologies carry worse prognoses than others. For example, bowel ischaemia has a particularly bad prognosis and carries a highly variable outcome depending on extent of bowel involved, that is global or segmental ischaemia^25^^,^^26^. The quality assessment tool used for assessing the methodological quality score of the reviewed papers (*Table 3*) showed a range from 50–83 per cent adherence to the items which equates a medium to high reliability^12^, with interventional studies scoring higher than observational studies on average.

**Table 3:** Quality Assessment Tool for Studies with Diverse Designs scores for all reviewed papers

Study no.	1	2	3	4	5	6	7	8	9	10	11	12	13	14	15	16
**Explicit theoretical framework**	3	3	3	3	3	3	3	3	3	3	3	3	3	3	3	3
**Statement of aims/objectives in main body of report**	3	3	3	3	3	3	3	3	3	3	3	3	3	3	3	3
**Clear description of research setting**	3	3	3	3	3	3	3	3	3	2	3	3	3	3	3	3
**Evidence of sample size considered in terms of analysis**	3	0	3	2	1	3	0	0	0	0	3	0	0	0	0	0
**Representative sample of target group of a reasonable size**	3	3	3	3	3	3	3	2	2	2	3	3	2	3	3	3
**Description of procedure for data collection**	3	3	3	3	1	3	3	2	2	2	3	2	2	2	3	3
**Rationale for choice of data collection tool(s)**	3	2	2	2	0	2	0	1	0	0	0	0	1	2	3	0
**Detailed recruitment data**	3	3	3	3	3	3	3	3	3	2	3	2	3	3	3	3
**Statistical assessment of reliability and validity of measurement tool(s) (quantitative only)**	0	0	0	0	0	0	0	0	0	0	0	0	0	0	0	0
**Fit between stated research question and method of data collection (quantitative)**	3	3	3	3	3	3	3	3	3	3	3	3	3	3	3	3
**Fit between stated research question and format and content of data collection tool, e.g. interview schedule (qualitative)**	N/A	N/A	N/A	N/A	N/A	N/A	N/A	N/A	N/A	N/A	N/A	N/A	N/A	N/A	N/A	N/A
**Fit between research question and method of analysis**	3	3	3	3	3	3	3	3	3	3	3	2	3	3	3	3
**Good justification for analytical method selected**	3	3	3	2	3	3	3	2	0	1	3	0	0	1	3	0
**Assessment of reliability of analytical process (qualitative only)**	N/A	N/A	N/A	N/A	N/A	N/A	N/A	N/A	N/A	N/A	N/A	N/A	N/A	N/A	N/A	N/A
**Evidence of user involvement in design**	0	0	0	0	0	0	0	0	0	0	0	0	0	0	0	0
**Strengths and limitations critically discussed**	2	2	2	3	2	3	3	2	0	2	3	0	2	2	3	0
**Score/42**	35	30	34	33	28	35	30	27	22	23	33	21	25	28	33	24
**Percentage**	83%	71%	81%	79%	67%	83%	71%	64%	52%	55%	79%	50%	60%	67%	79%	57%

Scores range from 0–3.

Looking forward, there is clearly a drive to improve patient outcomes and mortality by focusing and implementing change. In the UK, the National Emergency Laparotomy Audit has been in place for several years and has demonstrated a benefit to patients with improved outcomes in terms of mortality. The SMASH trial (Standardised perioperative management of patients operated with acute abdominal surgery in a high-risk setting) is an ongoing single-centre controlled trial by Timan and colleagues, due to be complete by 2026. This Swedish trial aims to determine whether standardized protocol-based perioperative management in emergency abdominal surgery leads to a better survival outcome. A short interval between the decision to perform surgery and the start of surgery is one of eight standardized preoperative measures^32^.

Despite the pragmatic approach to the inclusion criteria, this systematic review has demonstrated that there is limited high-quality evidence on the extent that interventions in the presurgery phases of acute laparotomy pathways benefit patients. Most studies agree that reducing overall time from presentation to surgery results in marked improvement in the perioperative mortality rate, but it is unclear which aspects of the pathway provide the greatest potential for gain. This review proposes a framework which allows standardization of how the presurgery aspects of the emergency laparotomy pathway are defined and assessed. The ‘symtoms2surgery’ framework could be incorporated into national mortality prediction and audit tools and in core outcome sets of perioperative studies concerning acute abdominal pathology across Europe.

## Funding

This study is supported by the National Institute for Health Research (NIHR) infrastructure at Leeds Teaching Hospital Trust and The University of Leeds. The views expressed are those of the authors and not necessarily those of the NHS, the NIHR or the Department of Health.

*Disclosure*. The authors declare no conflict of interest.

## Supplementary material

Supplementary material is available at *BJS Open* online

## Supplementary Material

zrab072_Supplementary_Data
