# Supplementary material for: Delay to surgery in acute perforated and ischaemic gastrointestinal pathology: a systematic review
Source: BJS Open. 2021 Sep 3;5(5):zrab072. doi: 10.1093/bjsopen/zrab072 (PMC8413368; doi:10.1093/bjsopen/zrab072)
Supplement: zrab072_Supplementary_Data [file zrab072_supplementary_data.docx]

**Search strategy**

| **“Keyword” Search Strategy for OVID (MEDLINE and EMBASE)** |
| --- |
| 1. Acute abdomen 2. Acute laparotomy 3. Emergency laparotomy 4. Emergency abdominal surgery 5. Acute abdominal pathology 6. Acute surgical abdomen 7. Acute abdominal pain 8. Perforated GI viscus 9. Perforated gastrointestinal viscus 10. Perforated viscus 11. Ischaemic GI viscus 12. Ischemic GI viscus 13. Ischaemic gastrointestinal viscus 14. Ischemic gastrointestinal viscus 15. Perforated bowel 16. Perforated peptic ulcer* 17. Perforated gastric ulcer 18. Perforated duodenal ulcer 19. Perforated closed loop obstruction 20. Colonic perforation 21. Diverticular perforation 22. Ischaemic bowel 23. Ischemic bowel 24. Ischaemic closed loop obstruction 25. Ischemic closed loop obstruction 26. Acute mesenteric ischaemia 27. Acute mesenteric ischemia 28. Acute ischaemic bowel 29. Acute ischemic bowel 30. Strangulated bowel 31. Delay 32. Time to theatre 33. Time to surgery 34. Operation 35. Surg* 36. Mortality 37. Morbidity 38. Mortality risk 39. Surviv* 40. Death 41. Length of stay 42. 1 OR 2 OR 3 OR 4 OR 5 OR 6 OR 7 OR 8 OR 9 OR 10 OR 11 OR 12 OR 13 OR 14 OR 15 OR 16 OR 17 OR 18 OR 19 OR 20 OR 21 OR 22 OR 23 OR 24 OR 25 OR 26 OR 27 OR 28 OR 29 OR 30 43. 31 OR 32 OR 33 OR 34 OR 35 44. 36 OR 37 OR 38 OR 39 OR 40 OR 41 45. 42 AND 43 AND 44 46. Limit 45 to English language 47. Limit 46 to Human studies 48. Limit 47 to year = ‘2004 – current’ |
